# Platelet-derived exosomes induce endothelial cell apoptosis through peroxynitrite generation: experimental evidence for a novel mechanism of septic vascular dysfunction

**DOI:** 10.1186/cc6133

**Published:** 2007-09-25

**Authors:** Marcela Helena Gambim, Alipio de Oliveira do Carmo, Luciana Marti, Sidney Veríssimo-Filho, Lucia Rossetti Lopes, Mariano Janiszewski

**Affiliations:** 1 Division of Rheumatology, University of São Paulo School of Medicine, Avenida Doutor Arnaldo, 455, 01246-903 – São Paulo – SP; 2 Instituto de Ensino e Pesquisa, Sociedade Beneficente Israelita-Brasileira Hospital Albert Einstein, Avenida Albert Einstein, 627 – Piso Chinuch, 05651-901 – São Paulo – SP; 3 Pharmacology Department, Biomedical Sciences Institute, University of São Paulo, Av. Prof. Lineu Prestes, 1524. Cidade Universitária "Armando de Salles Oliveira", 05508-900 – São Paulo – SP

## Abstract

**Introduction:**

Several studies link hematological dysfunction to severity of sepsis. Previously we showed that platelet-derived microparticles from septic patients induce vascular cell apoptosis through the NADPH oxidase-dependent release of superoxide. We sought to further characterize the microparticle-dependent vascular injury pathway.

**Methods:**

During septic shock there is increased generation of thrombin, TNF-α and nitric oxide (NO). Human platelets were exposed for 1 hour to the NO donor diethylamine-NONOate (0.5 μM), lipopolysaccharide (LPS; 100 ng/ml), TNF-α (40 ng/ml), or thrombin (5 IU/ml). Microparticles were recovered through filtration and ultracentrifugation and analyzed by electron microscopy, flow cytometry or Western blotting for protein identification. Redox activity was characterized by lucigenin (5 μM) or coelenterazine (5 μM) luminescence and by 4,5-diaminofluorescein (10 mM) and 2',7'-dichlorofluorescein (10 mM) fluorescence. Endothelial cell apoptosis was detected by phosphatidylserine exposure and by measurement of caspase-3 activity with an enzyme-linked immunoassay.

**Results:**

Size, morphology, high exposure of the tetraspanins CD9, CD63, and CD81, together with low phosphatidylserine, showed that platelets exposed to NONOate and LPS, but not to TNF-α or thrombin, generate microparticles similar to those recovered from septic patients, and characterize them as exosomes. Luminescence and fluorescence studies, and the use of specific inhibitors, revealed concomitant superoxide and NO generation. Western blots showed the presence of NO synthase II (but not isoforms I or III) and of the NADPH oxidase subunits p22^phox^, protein disulfide isomerase and Nox. Endothelial cells exposed to the exosomes underwent apoptosis and caspase-3 activation, which were inhibited by NO synthase inhibitors or by a superoxide dismutase mimetic and totally blocked by urate (1 mM), suggesting a role for the peroxynitrite radical. None of these redox properties and proapoptotic effects was evident in microparticles recovered from platelets exposed to thrombin or TNF-α.

**Conclusion:**

We showed that, in sepsis, NO and bacterial elements are responsible for type-specific platelet-derived exosome generation. Those exosomes have an active role in vascular signaling as redox-active particles that can induce endothelial cell caspase-3 activation and apoptosis by generating superoxide, NO and peroxynitrite. Thus, exosomes must be considered for further developments in understanding and treating vascular dysfunction in sepsis.

## Introduction

The concept of exosomes appeared with the description of the shedding process of the transferrin receptor by maturing reticulocytes [1]. Diverging from the idea of an accidental membrane fragmentation or from the apoptosis-associated bubbling of the plasma membrane, evidence accumulated during the past 5 years has revealed a very specific process of protein and lipid sorting that culminates with the generation of these small (about 100 nm in diameter) membrane vesicles [2]. Exosomes are released from dendritic cells [3], B lymphocytes [4], from different epithelial cell lines [5,6] and also from platelets [7]. They contain major histocompatibility complex class I and II molecules, cytosolic chaperone proteins, subunits of trimeric G proteins, cytoskeletal proteins, annexins, integrins, enzymes, and elongation factors [8]. Several of these proteins have known functions in fusion, adhesion and biosynthetic processes, but most have yet to be assigned specific roles in exosome formation and function. Initial studies demonstrated co-stimulatory as well as suppressive effects on immunological signaling. Recent studies have led to the hypothesis that exosome interchange may in fact represent a novel pathway of intercellular communication [8,9]. Nevertheless, there are as yet no experimental indications of how exosomes interact with their target cells. The exosomes could fuse with the plasma membrane, they could be endocytosed, or they could merely attach to the cell surface, modifying transmembrane signaling pathways.

Endothelial activation is physiologically important in the context of the inflammatory response as well as pathophysiologically in ischemia/reperfusion, sepsis, and early atherosclerosis [10]. In view of the importance of endothelial function in cardiovascular homeostasis, the mechanisms underlying endothelial activation and the development of endothelial dysfunction are of great interest. A large body of evidence indicates that the generation of reactive oxygen species (ROS) and reactive nitrogen species (RNS), both within endothelial cells and in the adjacent milieu, has a major role in endothelial activation and dysfunction. Mitochondrial ROS generation seems to have a major role in modulating physiological responses to oxygen tension and flow variations [11,12]. In contrast, under pathological conditions there is evidence that reinforces the role not only for mitochondria but also for the two main enzymatic sources of ROS and RNS within the vascular tissue: the superoxide-generating NADPH oxidases and the NO synthases [13-15]. In this context, platelets are known to express both enzymes with corresponding activities, although a clear role for platelet-derived ROS in vascular dysfunction has not been assigned [16,17].

In previous work we have shown that, in sepsis, platelet-derived microparticles similar to exosomes can be recovered from plasma and that incubation of these microparticles with vascular cells induces apoptosis *in vitro *through a NADPH oxidase-dependent pathway [18]. Here we further investigated this mechanism, definitively characterizing these microparticles as exosomes, and revealing NO and lipopolysaccharide (LPS) as possible triggers for their release. In addition, we show that exosome-generated peroxynitrite induces endothelial cell caspase-3 activation followed by apoptosis, revealing a putative novel pathway for platelet-induced septic vascular dysfunction.

## Materials and methods

### Cell culture

The established endothelial cell line derived from rabbit aorta characterized by Venter and Buonassisi [19] was a gift from Jose Eduardo Krieger (Heart Institute, University of São Paulo School of Medicine, São Paulo, Brazil). Cells were maintained in Ham's F12 medium supplemented with 10% (v/v) heat-inactivated fetal bovine serum (Invitrogen Brasil Ltda, São Paulo, Brazil) and allowed to grow to about 80% confluence. For 24 hours before use, cells were kept with 1% serum-supplemented medium to cause phase arrest.

### Obtaining platelet-derived exosomes from septic patients

Blood samples (40 ml) were collected from 12 patients admitted to the intensive care unit of the Hospital Israelita Albert Einstein (São Paulo, Brazil), with early (24 hours) diagnosis of septic shock, as defined in accordance with the criteria of the American College of Chest Physicians and the Society of Critical Care Medicine [20]. Patients were not on any antiplatelet or anti-inflammatory drug. The study was approved by the Institutional Ethics Board. Clinical data about septic patients and control subjects are given in Table 1.

**Table 1:** Clinical data for septic patients and healthy controls

Characteristic	Patients (*n *= 12)	Controls (*n *= 10)	*P*
Age	58.3 ± 21	39.5 ± 13	0.02
Platelet count/ml	(187 ± 45) × 10^6^	(270 ± 116) × 10^6^	0.03
Exosome mg protein/sample	9.6 ± 3.9	10.6 ± 4.5	0.56
Infection			
Gram-negative	6	n.a.	
Gram-positive	2	n.a.	
*Candida*	1	n.a.	
Unidentified	3	n.a.	
Site of origin			
Respiratory	7	n.a.	
Blood	2	n.a.	
Urinary	1	n.a.	
Peritonitis	1	n.a.	
Trauma	1	n.a.	
Neutrophil count/ml	(12.1 ± 5.7) × 10^3^	(5.6 ± 1.5) × 10^3^	0.002
Dysfunction			
Shock	8	n.a	
Respiratory	8	n.a.	
Renal	3	n.a.	
Hepatic	1	n.a.	

n.a., not applicable.

Blood was collected in centrifuge tubes containing 10.5 mM trisodium citrate and was processed immediately. Initial procedures were performed at room temperature (between 20–25°C) to avoid artifactual platelet activation. Cells, platelets, and large debris were pelleted by centrifugation at 3,000 *g *for 10 minutes. Phenylmethanesulfonyl fluoride (3 mM), aprotinin (1 g/ml), and pepstatin (1 g/ml) as protease inhibitors were added to the supernatant, which was then sequentially filtered through 1.0, 0.45, and 0.22 μm nylon filters to remove platelets, cellular fragments, and apoptotic bodies. The remaining cell-free plasma was collected over ice and ultracentrifuged at 100,000 *g *for 90 minutes at 4°C. The pellet, containing exosomes, was first washed with PBS containing 0.1 mM EDTA to avoid contamination with plasma proteins, and then resuspended in 250 μl of PBS. The total exosome mass obtained was 9.6 ± 3.9 mg protein per sample. In previous work we have shown that this exosome population displayed almost exclusively platelet markers [18].

### Obtaining platelet-derived exosomes from healthy volunteers

Blood (40 to 50 ml) was collected from healthy volunteers who had not taken any medication known to interfere with platelet function within the previous 2 weeks. The blood was drawn into tubes containing acid citrate dextrose anti-coagulant (3.8 mM citric acid, 7.5 mM trisodium citrate, 125 mM dextrose, 1.8 ml anti-coagulant per 8.1 ml of whole blood). Platelet-rich plasma was first obtained by centrifugation at 800 *g *for 5 minutes at 20°C, and subsequently leukocytes were removed through a commercial filter system (Pall Corporation, East Hills, NY, USA). Plasma-free platelet suspensions were obtained by centrifugation of platelet-rich plasma at 800 *g *for 15 minutes at 20°C, and the resultant pellet was resuspended in 5 ml of Krebs-HEPES buffer (in mM: NaCl 99, KCl 4.7, MgSO_4 _1.2, KH_2_PO_4 _1, CaCl_2 _1.9, NaHCO_3 _25, glucose 11.1, and sodium HEPES 20).

Plasma-free platelet suspensions were incubated with agonist or with saline control (154 mM NaCl in water) for 1 hour as indicated, and the reaction was slowed down by placing samples on ice. Samples were centrifuged (800 *g *for 15 minutes) to obtain the platelet pellet fraction. The supernatant was further centrifuged (17,500 *g *at 30 minutes) to obtain the microvesicle fraction, and the supernatant from that microvesicle fraction was filtered sequentially through 0.45 and 0.22 μm low-protein-binding nylon membranes. The filtered product was further centrifuged (100,000 *g *for 90 minutes) to obtain the exosome pellet. All pellets were resuspended in 250 μl of PBS. The total exosome mass obtained was 10.6 ± 4.5 mg of protein per sample.

### Creation of a model resembling platelet-derived exosomes from septic patients

Sepsis and septic shock can be viewed as a state of immuno-inflammatory imbalance in response to an infection. Different models have been validated to simulate sepsis under *in vivo *or *in vitro *conditions, such as exposure to LPS or TNF-α. LPS is a component of the bacterial cellular wall known to stimulate the innate immuno-inflammatory response through Toll-like receptors present in leukocytes, dendritic cells, and endothelial cells [21]. TNF-α is a cytokine released in the early phases of the septic response and is believed to have a central role in its initial steps, promoting the further release of other inflammatory and anti-inflammatory cytokines and altering the vascular wall, leading to increased endothelial stickiness and permeability [22]. It is also well known that part of the vascular dysfunction arising during the clinical course of septic shock is due to an enhanced production of nitric oxide (NO) [23]. We therefore decided to stimulate platelets with those agents to create a suitable model of platelet exosome generation, similar to those found in septic patients. Platelets were incubated for 1 hour at room temperature with 100 ng/ml LPS, or 40 ng/ml human TNF-α, or with the NO donor diethylamine-NONOate (0.5 μM). Platelets incubated with 250 μl of saline or with 5 IU/ml thrombin were used as controls.

To generate apoptotic bodies, which served as controls for phosphatidylserine-exposing particles, apoptosis was induced in rabbit endothelial cells by treatment with ultraviolet radiation [18,24]. In brief, after cells reached about 80% confluence on Petri dishes, culture medium was replaced with PBS and cells were irradiated for 30 minutes with ultraviolet radiation with a TUV 15 W/G15 T8 lamp (Philips, The Netherlands). After irradiation, fresh medium was added and cells were cultured for a further 24 hours. Supernatant medium was collected and centrifuged successively at 1,200 *g *and 10,000 *g *to pellet cells and large debris and finally at 100,000 *g *to collect apoptotic bodies.

### Detection of reactive species

Measurements of the generation of reactive species were all performed in a FARCyte plate reader (Amersham Biotech, Buckinghamshire, UK). Exosomes were resuspended in 100 μl of Krebs-HEPES buffer at a constant 100 μg/ml concentration. Luminescent or fluorescent probes were added 15 minutes before measurements started, and samples were equilibrated while being protected from light.

The luminescent probes lucigenin and coelenterazine were first used to detect the generation of ROS. The concentration of lucigenin and coelenterazine used (5 μM each) minimized the generation of artifactual readings, as shown previously [25]. Reactions were started by adding NADPH (0.1 mM) for the lucigenin assay and NADPH (0.1 mM) plus L-arginine (1 μM) for coelenterazine. Luminescence signals were measured in solid white plates, with the integration time set to 1,000 ms, without attenuation; background was automatically subtracted from all measurements. To compare the generation of ROS in exosomes with that in whole platelets, lucigenin and coelenterazine assays were performed with 10^8 ^platelets/ml and results were corrected to protein content. Luminescent counts are presented as relative luminescence units (RLU)/min per mg of protein.

To better characterize the generation of reactive species, 2',7'-dihydrodichlorofluorescein diacetate (DCHF; 10 mM) for ROS [25] and 4,5-diaminofluorescein diacetate (DAF; 10 mM) for RNS [26] were used. Measurements were performed in the presence of NADPH (0.1 mM) with or without L-arginine (1 μM) for DCHF, and in the presence of L-arginine for DAF.

Further studies to characterize the source or type of reactive species were performed in the presence of specific inhibitors or quenchers such as L-NMA (*N*^G^-methyl-L-arginine acetate; 5 mM), L-NAME (*N*^ω^-nitro-L-arginine methyl ester; 1 μM) and D-NAME (*N*^ω^-nitro-D-arginine methyl ester; 1 μM), urate (1 μM), the membrane-permeable superoxide dismutase mimetic Mn(III) tetrakis (4-benzoic acid) porphyrin chloride (SOD mimetic; 10 μM; Oxys Research, Portland, OR, USA), and the specific NADPH oxidase inhibitory peptide gp91ds-tat (10 μM) [27].

### Flow cytometry

For flow cytometry analysis, we used aliquots of exosome or apoptotic body suspensions with 200 μg of particle protein/ml. To identify specific epitopes, aliquots were incubated with fluorescein 5(6)-isothiocyanate (FITC) or R-phycoerythrin-conjugated antibodies directed to specific membrane antigens at 1 μg/ml final concentration (BD Biosciences, San Jose, CA, USA), namely CD9, CD63, and CD81 (molecules from the tetraspan co-activator family, which characterize exosomes) [4,8], and with annexin V-FITC conjugate in a calcium-containing binding buffer. Binding of annexin V indicates the exposure of phosphatidylserine on the particle surface. In contrast to signaling exosomes, apoptotic bodies are known to expose large amounts of phosphatidylserine [24]. Samples were acquired in a FACScan flow cytometer and analyzed with CellQuest software (Becton Dickinson, San Jose, CA, USA). Non-specific signals were inhibited by the addition of normal species serum. Binding of specific antibodies was corrected with identical concentrations of control IgG antibodies. Thresholds were set to correct for nonspecific antibody binding or fluorescence.

Because exosomes are, on average, too small for cytometry analysis, we believe that our data correspond to aggregates formed after ultracentrifugation. For this reason we did not attempt to perform any specific quantification.

### Electron microscopy

Pellets of exosomes obtained from platelets were fixed under 2.0% glutaraldehyde in 0.1 M sodium cacodylate for at least 2 hours and postfixed with 2% osmium tetroxide in 10.56% sucrose for 2 hours and finally incubated with 0.5% uranyl acetate and 10.56% sucrose overnight. Pellets were then dehydrated and embedded in Spurr resin. Ultrathin sections 70 to 80 nm thick were cut on an ultramicrotome (Leica Ultracut R, Leica Microsystems GmbH, Wetzlar, Germany), picked up on copper grids and stained for contrast with 1% uranyl acetate and 1% lead citrate. Specimens were examined with a transmission electron microscope (Jeol Electric 1010; Jeol Ltd, Tokyo, Japan), operated at 80 kV.

### Quantification of apoptosis

Annexin V was used to detect apoptosis [28]. In brief, rabbit endothelial cells were grown on six-well plates as described. For 24 hours before use, cells were kept with 1% serum to cause phase arrest. A volume of exosome suspension equivalent to 100 μg of protein was added to each well (final protein concentration per well 400 μg/ml) and left to incubate for 30 minutes. Some experiments were performed after incubation with the membrane-permeable SOD mimetic (10 μM), with urate (1 μM), or with L-NAME (1 μM). After incubation, cells were washed, fresh medium was added. After 1 hour, cells were washed with ice-cold PBS and removed from the plates with 1% trypsin, followed by a short centrifugation and resuspension in calcium-containing binding buffer at a 10^6 ^cells/ml into Eppendorf vials. Annexin V-FITC was added to a final concentration of 100 ng/ml, and the cells were incubated in the dark for 10 minutes and then washed again with PBS. Propidium iodide (30 μl) was added before analysis. Cells were spread on clean slides, covered with glass coverslips, and immediately examined under fluorescence microscopy. From three high-power fields per sample, a minimum of 200 cells were counted. Cells were considered apoptotic when membrane-bound annexin-FITC fluorescence was positive and nuclear staining with propidium iodide (evidence of late apoptosis or necrosis) was negative. Results are expressed as apoptotic cells per 100 cells.

### Caspase-3 activation

Rabbit endothelial cells were cultured on six-well plates to 80 to 90% confluence as described. Cells were kept in 1% serum for 24 hours before use. A volume of microparticle suspension equivalent to 100 μg of protein was added to each well (final protein concentration per well 400 μg/ml) and incubated for 30 minutes. Some experiments were performed after incubation with the membrane-permeable SOD mimetic (10 μM) or with L-NAME (1 μM). Exposure to TNF-α (50 ng/ml) was used as a positive control for caspase-3 activation. After incubation, plates were kept on ice. Cells were washed with ice-cold PBS and lysed with Nonidet lysis buffer containing Tris/HCl (20 mM, pH 7.4), NaCl (150 mM), Na_4_P_2_O_7 _(10 mM), leupeptin (1 μg/ml), pepstatin (1 μg/ml), phenylmethylsulfonyl fluoride (3 mM), and Nonidet P40 (1% v/v), placed on ice for 10 minutes, and centrifuged at 10,000 *g *for 10 minutes. The activity of caspase-3 was measured at 405 nm with a Caspase-3 Colorimetric Detection Kit (Assay Designs, Ann Arbor, MI, USA) in accordance with the manufacturer's instructions.

### Western blots

Exosome protein (40 μg), leukocyte and endothelial cell lysate (used as a positive control) were subjected to separation by SDS-PAGE and transferred to nitrocellulose. Equal separation and transference of the samples were confirmed by Ponceau staining during the preparation of membranes. Membranes were incubated with antibodies directed to the NADPH oxidase cytochrome *b*_558 _components p22^phox^, Nox1, and Nox2 (gp91^phox^) (1:1,000 dilution; Santa Cruz Biotechnology, Santa Cruz, CA, USA) or to inducible nitric oxide synthase (NOS), endothelial NOS or neuronal NOS (1:1,000 dilution; Chalbiochem, EMD Chemicals, San Diego, CA, USA) followed by horseradish peroxidase-conjugated secondary antibody (1:5,000 dilution; Santa Cruz Biotechnology) and developed with the Chemiluminescence-Phototope-HRP (horseradish peroxidase)-conjugated Detection Kit (New England Biolabs, Beverly, MA, USA) as specified. Results are representative of at least three similar experiments.

### Data analysis

Data shown are means ± SD of three or more similar experiments. Comparisons between groups were performed by one-way analysis of variance followed by a Student–Newman–Keuls test at *P *< 0.05 significance level.

## Results

### Flow cytometry

**Figure 1:**
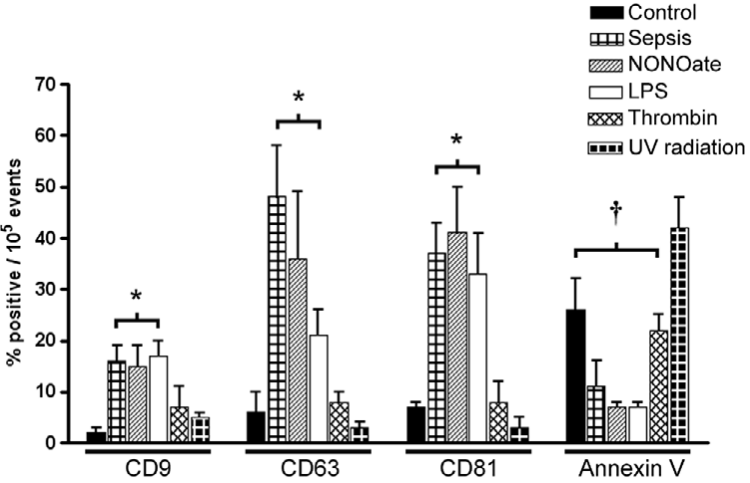
Tetraspan protein enrichment characterizes exosomes. The graph shows the percentage of positive events per 100,000 counts as analyzed by flow cytometry. Values are corrected for background and non-specific antibody binding. Exosomes obtained from septic patients as well as from platelets activated by the nitric oxide donor diethylamine-NONOate (NONOate; 0.5 μM) or lipopolysaccharide (LPS; 100 ng/ml) expose larger amounts of tetraspan protein family members CD9, CD63, and CD81, and less phosphatidylserine (as assessed by annexin V staining) than particles obtained from platelets treated only with saline (Control) or thrombin (5 IU/ml) or from apoptotic endothelial cells (apoptosis). Results are means ± SD. For each bar, *n *= 4 samples. *P < 0.05 versus control, ^†^*P *< 0.05 versus apoptotic bodies (apoptosis). UV, ultraviolet.

Exosomes are known to expose several different markers related to their cellular origin and putative functions. Phosphatidylserine is typically not exposed, differentiating exosomes from apoptotic bodies or cellular debris. In contrast, proteins of the tetraspan family are considered to be specifically sorted during exosome generation. As shown in Figure 1, flow cytometry analysis clearly divided the exosomes in two groups: those obtained from platelets stimulated with either the NO donor diethylamine-NONOate or LPS, and those recovered from platelets exposed to saline (control), thrombin, or TNF-α (not shown).

Exosomes in the former group, which are similar to those recovered from septic patients, exposed large amounts of the tetraspan family members CD9, CD63, and CD81 and exhibiting low binding of annexin V. Exosomes in the latter group were similar to the apoptotic bodies with lower tetraspan exposure and higher annexin V binding capability.

### Electron microscopy

**Figure 2:**
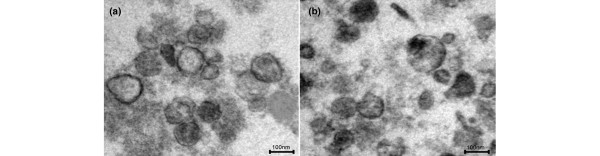
Electron microscopy reveals the structure of exosomes. Images obtained from the exosome population generated by platelets exposed to diethylamine-NONOate **(a) **and to thrombin **(b) **reveal rounded membranaceous structures measuring on average less than 150 nm. It is noteworthy that exosomes from platelets stimulated with diethylamine-NONOate have a more regular surface than those generated by platelets exposed to thrombin. Scale bars, 100 nm; original magnification ×60,000.

Electron microscopy (Figure 2) revealed typical saucer-like structures with a diameter of 100 to 200 nm, which correspond to exosomes. Figure 2a shows exosomes derived from platelets exposed to diethylamine-NONOate, and Figure 2b exosomes from platelets exposed to thrombin.

### Generation of reactive species

**Figure 3:**
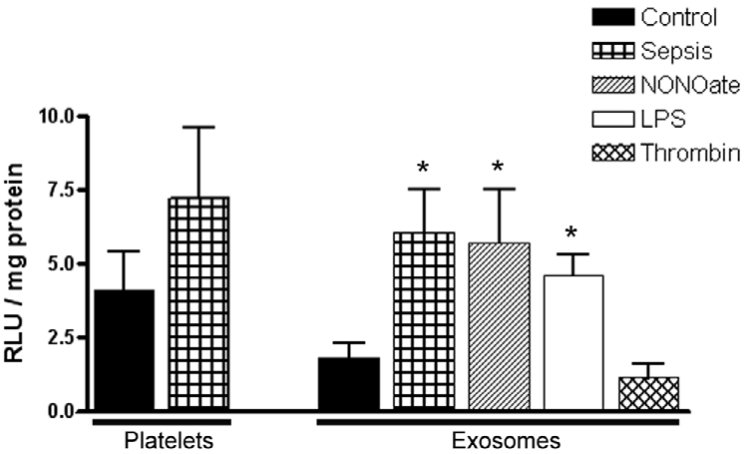
Lucigenin luminescence: exosomes from platelets exposed to NO or LPS are similar to septic exosomes. The graph represents NADPH-dependent lucigenin (5 μM) chemiluminescence above background. Exosomes (10 μg protein content) obtained from platelets exposed to the nitric oxide donor diethylamine NONOate (NONOate; 0.5 μM) or lipopolysaccharide (LPS; 100 ng/ml) generate reactive oxygen species in a similar fashion to exosomes obtained from septic patients, whereas particles obtained from platelets exposed to saline (control) or thrombin (5 IU/ml) have very low activity. For comparison, luminescence obtained with platelets from healthy (control) and septic subjects are displayed. Results normalized for sample protein concentration are means ± SD of three or more experiments. *P < 0.05 versus control.

Preliminary measurements of ROS-generating activity, performed with lucigenin, revealed that the redox activity of exosomes paralleled the surface characteristics disclosed by flow cytometry analysis. As seen in Figure 3, exosomes obtained from platelets exposed to LPS or to the NO donor generated ROS in a similar manner to that of exosomes from septic patients, whereas exosomes obtained from platelets exposed to saline (control), thrombin, or TNF-α (not shown) generated very small amounts of ROS. Intact platelets generated substantially higher luminescent signals than exosomes. Platelets from septic patients also displayed higher ROS generation than controls. The SOD mimetic and L-NAME had a similar inhibitory effect on whole-platelet ROS generation.

**Figure 4:**
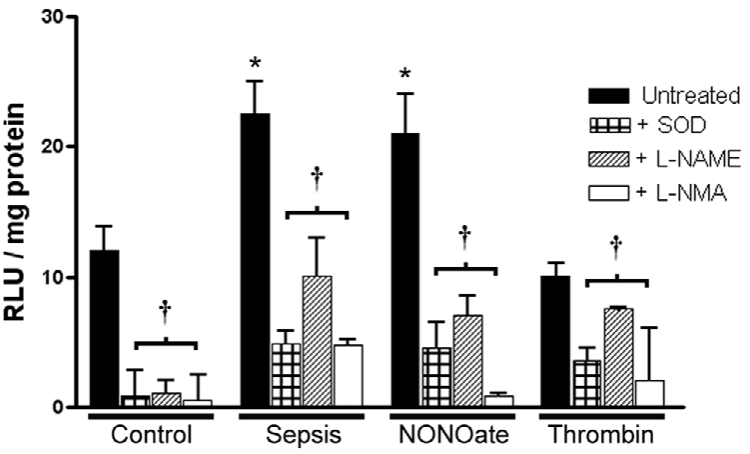
Coelenterazine luminescence triggered by exosomes suggests the presence of reactive oxygen and nitrogen generation. The graph represents exosome coelenterazine (5 μM) luminescence above background. Exosomes were incubated with NADPH and L-arginine. Exosomes (10 μg protein content) obtained from platelets exposed to the nitric oxide donor diethylamine NONOate (NONOate; 0.5 μM) or lipopolysaccharide generate reactive oxygen species in a similar fashion to exosomes obtained from septic patients, whereas particles obtained from platelets exposed to saline (control) or thrombin have very low activity. Luminescent signals were consistently inhibited by the addition of the superoxide dismutase mimetic Mn(III) tetrakis (4-benzoic acid) porphyrin chloride (SOD, 10 μM) and by the NO synthase inhibitors L-NMA (*N*^G^-methyl-L-arginine acetate; 5 mM), or *N*^ω^-Nitro-L-arginine methyl ester (L-NAME; 1 mM), suggesting the generation of reactive oxygen species and reactive nitrogen species by the exosomes. Results are means ± SD of seven experiments. *P < 0.05 versus control, ^†^*P *< 0.05 versus untreated. RLU, relative luminescence units.

To characterize the exosome redox profile better, measurements with the luminescent probe coelenterazine were also performed (Figure 4). Results were similar to those obtained with lucigenin. Furthermore, coelenterazine is known to react with both superoxide and peroxynitrite. The SOD mimetic and the NO synthase inhibitors L-NAME and L-NMA significantly inhibited the luminescent signals, suggesting that platelet exosomes are able to generate both superoxide and NO. Controls with D-NAME did not show any significant decrease in signal.

The fluorescent probes DCHF and DAF were used for further clarification of the nature of ROS generated by the exosomes. DCHF is believed to react with hydrogen peroxide, whereas detection of superoxide radical with DCHF is still not clear. DCFH can also be oxidized by peroxinitrite [25]. In contrast, DAF is considered a specific probe for RNS, such as NO or peroxynitrite.

**Figure 5:**
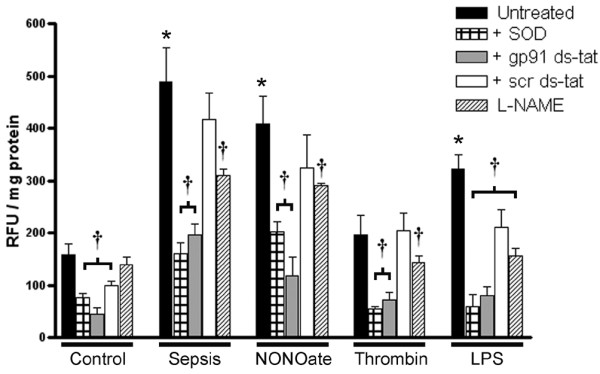
NADPH oxidase and uncoupled NO synthase are sources of reactive species from platelet-derived exosomes. Exosomes from septic patients, as well as exosomes induced with the nitric oxide donor diethylamine NONOate (NONOate; 0.5 μM) and lipopolysaccharide (LPS) caused enhanced 2',7'-dichlorofluorescein diacetate (10 mM) fluorescence (after the addition of 100 μM NADPH), which was significantly inhibited by the membrane-permeable superoxide dismutase mimetic Mn(III) tetrakis (4-benzoic acid) porphyrin chloride (SOD) or by the NADPH oxidase-blocking peptide gp91 ds-tat (10 μM), confirming the role of a superoxide-generating NADPH oxidase. *N*^ω^-nitro-D-arginine methyl ester (L-NAME) decreased the fluorescent signals, suggesting a role for uncoupled nitric oxide synthase in superoxide generation. The scrambled peptide (scr ds-tat) used as a control for gp91 ds-tat shows a non-significant residual inhibitory effect. Results are means ± SD of five experiments for each group. *P < 0.05 versus control, ^†^*P *< 0.05 versus untreated. RFU, relative fluorescence units.

**Figure 6:**
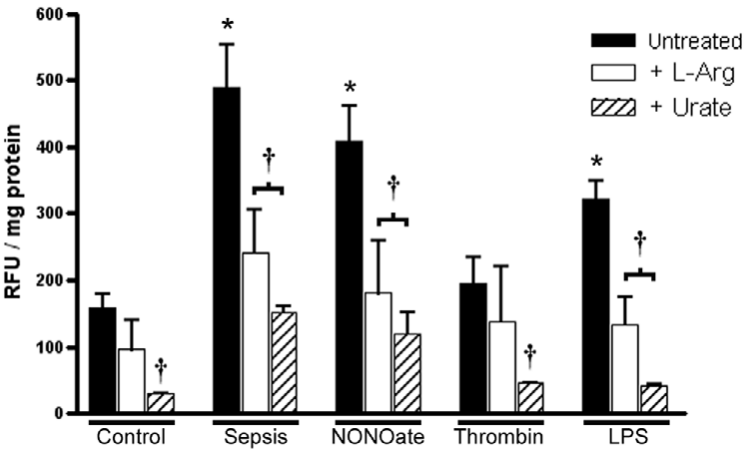
Platelet-derived exosomes may generate peroxynitrite. The graph shows a decrease in 2',7'-dichlorofluorescein diacetate signals after the addition of L-arginine (1 mM), further suggesting a role for uncoupled nitric oxide synthase in superoxide generation. In contrast, the inhibitory effect of urate addition strongly suggests the involvement of peroxynitrite oxidation. Results are means ± SD of five experiments for each group. *P < 0.05 versus control, ^†^*P *< 0.05 versus untreated. NONOate, diethylamine NONOate; RFU, relative fluorescence units.

Figure 5 shows clearly that exosomes obtained from septic patients and from platelets stimulated with the NO donor diethylamine-NONOate or LPS generate large amounts of ROS, whereas exosomes from non-stimulated platelets (control) or from platelets exposed to thrombin do not possess this activity. DCHF signals were inhibited by the SOD mimetic, suggesting that superoxide could be involved. In a previous study we showed that exosomes from septic patients possess a superoxide-generating activity that is not inhibited by catalase (a hydrogen peroxide scavenger), fluconazol (a cytochrome P450 inhibitor) or by oxypurinol (a xanthine oxidase inhibitor), but sensitive to phenylarsine oxide and diphenylene iodonium, two well-known inhibitors of NADPH oxidase. To characterize the source of superoxide better, we performed experiments with the specific NADPH oxidase inhibitor peptide gp91ds-tat [27], which greatly decreased the DCHF fluorescence of exosomes compared with the scrambled peptide. These results indicate the participation of a Nox-based NADPH oxidase. Recent studies suggest that uncoupling of the NO synthase could also be a significant source of superoxide in the vascular milieu [11]. In fact, the addition of L-NAME, known to block not only the NO generation but also superoxide generation from uncoupled NO synthases, caused a 40% inhibition of DCHF fluorescence. In addition, supplementation with L-arginine (Figure 6), which may favor recoupling of the NO synthase, resulted in a similar decrease in DCHF signals, suggesting that electron transfer was redirected to NO synthesis. Finally, considering the coexistence of active NADPH oxidase and NO synthase, we postulate a role for peroxynitrite as a major oxidating species in this system, because the addition of urate abolished these effects (Figure 6).

**Figure 7:**
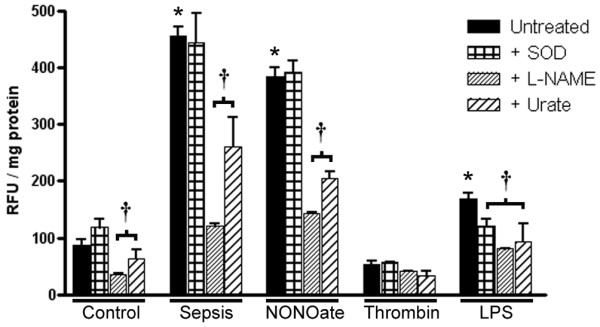
Exosomes generate reactive nitrogen species. The graphic shows 4,5-diaminofluorescein diacetate (10 mM) fluorescence of exosomes incubated with L-arginine. The membrane-permeable superoxide dismutase mimetic Mn(III) tetrakis (4-benzoic acid) porphyrin chloride (SOD) had no inhibitory effect, whereas *N*^ω^-nitro-D-arginine methyl ester (L-NAME) and urate caused a significant decrease in fluorescent signals, suggesting the generation of reactive nitrogen species by exosomes, more importantly by septic exosomes and by exosomes induced with nitric oxide or lipopolysaccharide (LPS). Results are means ± SD of four experiments for each group. *P < 0.05 versus control, ^†^*P *< 0.05 versus untreated. RFU, relative fluorescence units.

Figure 7 shows the results obtained with DAF. Exosomes from platelets exposed to the NO donor or to LPS had a similar activity profile to those from platelets obtained from septic patients, whereas exosomes from platelets exposed to thrombin or saline had low redox activity. Furthermore, DAF signals could be significantly decreased by the NO synthase inhibitor L-NAME and by the peroxynitrite scavenger urate, but not by the SOD mimetic. To investigate the source of NO, preliminary experiments were performed with addition of the dication chelator EDTA (1 mM) in probe buffer. Although no specific signal inhibition was noted, DAF signals became highly variable. Interference with intermediate reactions involved in signal generation was hypothesized. To clarify the situation, experiments were performed with calcium-containing or calcium-free Krebs-HEPES buffer. Under these conditions, DAF signals were not affected at all, indicating the existence of an active calcium-independent (inducible) NO synthase.

### Western blot analysis

**Figure 8:**
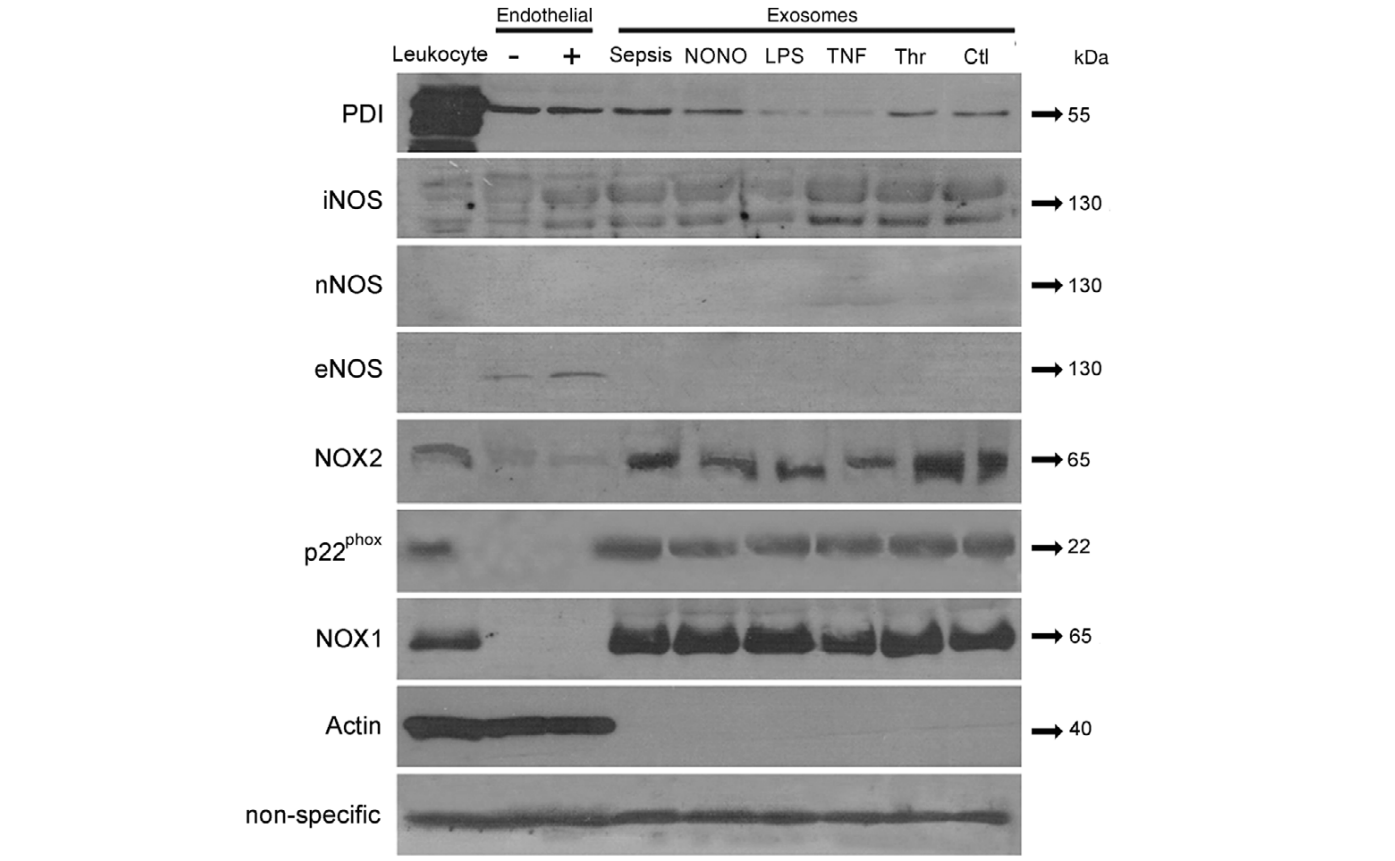
Platelet-derived exosomes possess NADPH oxidase and nitric oxide synthases. Representative Western blot images of exosomes from different origins (septic platelets (sepsis), platelets exposed to diethylamine-NONOate (NONO), lipopolysaccharide (LPS), TNF-α, thrombin (Thr) and saline (Ctl)) were subjected to SDS-PAGE and exposed to antibodies directed to the different nitric oxide synthase (NOS) isoforms: neuronal (nNOS), inducible (iNOS) and endothelial (eNOS), to the NADPH oxidase regulatory protein protein disulfide isomerase (PDI), to the NADPH oxidase membrane-bound subunit isoforms Nox 1 and Nox2, and to the NADPH oxidase membrane component p22^phox^. Leukocytes were used as positive controls for iNOS, and NADPH oxidase components, endothelial cells activated (+) or not (-) with LPS were used as controls for eNOS/iNOS expression. Results shown are representative of at least three different experiments.

Figure 8 summarizes the results of a Western blot analysis of the exosomes. As expected from the functional results, we were able to identify the presence of type II NO synthase but not that of types I or III. Furthermore, we were able to identify the subunits p22^phox^, Nox1, and Nox2 of the NADPH oxidase, as well as its regulatory protein protein disulfide isomerase (PDI). A non-specific protein staining can also be seen, which confirms equal gel loading between samples.

### Apoptosis

**Figure 9:**
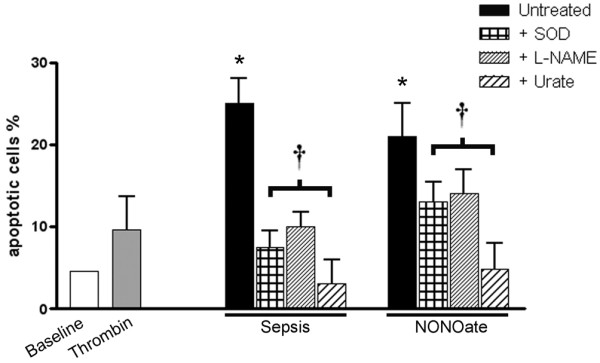
Nitric oxide-induced and septic platelet-derived exosomes cause ROS/RNS-dependent apoptosis in endothelial cells. Exosomes obtained from septic patients or from platelets exposed to a nitric oxide (NO) donor (diethylamine-NONOate; NONOate) cause a twofold to threefold increase in apoptosis rates of rabbit endothelial cells compared with exosomes from platelets exposed to saline (not shown) or thrombin. The membrane-permeable superoxide dismutase mimetic Mn(III) tetrakis (4-benzoic acid) porphyrin chloride (SOD; 10 mM), the NO synthase inhibitor *N*^ω^-nitro-L-arginine methyl ester (L-NAME; 1 mM), or the peroxynitrite scavanger urate (1 mM) reversed the proapoptotic activity of exosomes. Results are means ± SD of six experiments for each group. *P < 0.05 versus control, ^†^*P *< 0.05 versus untreated. ROS, reactive oxygen species; RNS, reactive nitrogen species.

To verify a physiological or pathophysiological role for platelet-derived exosomes, we exposed cultured endothelial cells to the different types of exosome. As seen in Figure 9, exosomes obtained from platelets exposed to thrombin had no effect on basal endothelial cell apoptotic rates (baseline). In addition, exosomes from platelets exposed to saline did not show any effect on endothelial apoptosis rate (data not shown). In contrast, exosomes from septic patients and exosomes from platelets exposed to an NO donor showed a twofold to threefold increase in apoptotic rates. This effect was heat sensitive and was fully inhibited by the SOD mimetic, the NO synthase inhibitor, and urate. These results suggest, in fact, a role for the ROS and RNS generated by enzymatic sources in the exosomes.

**Figure 10:**
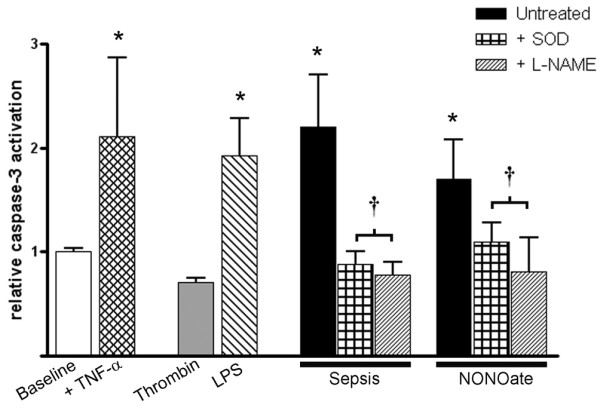
Exosomes cause reactive oxygen species/reactive nitrogen species-dependent caspase-3 activation in endothelial cells. Exosomes obtained from platelets exposed to saline (not shown) or thrombin did not cause caspase-3 activation above baseline in rabbit endothelial cells. In contrast, exosomes from septic patients (sepsis) or from platelets exposed to lipopolysaccharide (LPS) or a nitric oxide donor (diethylamine-NONOate; NONOate) caused a doubling of caspase-3 activation over baseline, similar to the activation obtained by direct exposure of endothelial cells to 40 ng/ml TNF-α (+TNF-α). The membrane-permeable superoxide dismutase mimetic Mn(III) tetrakis (4-benzoic acid) porphyrin chloride (SOD) and *N*^ω^-nitro-L-arginine methyl ester (L-NAME) completely blocked exosome-triggered caspase-3 activation. Results are means ± SD of three experiments for each group. *P < 0.05 versus control, ^†^*P *< 0.05 versus untreated.

Caspase-3 is one central step in the apoptotic cascade, and it is well known to be redox sensitive [29-31]. To verify whether exosome-induced apoptosis could be related to caspase-3 activation, we exposed endothelial cells to various exosome preparations and measured caspase-3 activation colorimetrically. Figure 10 summarizes the results, revealing that exosome-triggered caspase-3 activation paralleled increased apoptosis rates in endothelial cells. In addition, we demonstrated that caspase-3 activation is clearly dependent on the generation of superoxide or NO. Exosomes obtained from platelets exposed to saline did not show any significant effect (data not shown).

## Discussion

A basic role for exosomes in intercellular communication implies that the cell of origin controls their content. In this respect, it has been suggested that different agents are able to induce the release of phenotypically distinguishable platelet microparticles *in vitro *[32]. More recently, studies have clearly demonstrated that a specific protein sorting takes place during exosome formation from reticulocytes, from B cells, and from mononuclear blood cells, promoting the generation of raft-like domains, with a clear structure–function relationship [33]. In fact, one of the initial findings of our study was the confirmation that platelets secrete exosome-like particles with different characteristics after various stimuli: exosomes generated from platelets exposed to NO donors or LPS are quite similar to those found in septic patients as regards protein content, phosphatidylserine exposure, and redox activity, whereas platelets exposed to thrombin or TNF-α release clearly distinct particles. Furthermore, we found in the platelet-derived exosomes, both from septic shock patients and from platelets stimulated with LPS or NO, a high content of PDI. Interestingly, blood mononuclear cells subjected to heat shock specifically direct heat shock protein 70 (hsp70) to exosomes [34]. PDI, much like hsp70, is a chaperone, associated with protein transport from the endoplasmic reticulum to the membrane, and it is also closely related to the redox equilibrium of vascular cells. Recently it has been shown that PDI modulates NADPH oxidase in vascular smooth muscle cells [35]. This leads to hypotheses about the role of PDI (as well as other chaperones) in specific protein sorting in exosomes.

The mechanisms regulating the secretory process of exosomes are as yet completely unknown. They emerge from an intracytoplasmic membrane complex known as multivesicular bodies, which can be understood as a processing compartment for internalized proteins, subjected to the influence of the trans-Golgi network. Regulation of specific protein sorting to the multivesicular bodies has been explored better and apparently depends on lipid signaling involving phosphadylinositol kinases and ubiquitination [36]. In contrast, only one recent study suggested a regulatory pathway for secretion from exosomes, revealing that the inhibition of diacylglycerol kinase-α (DGK-α) in T lymphocytes increased the secretion of proapoptotic exosomes [37]. Inhibition of DGK isoforms allows full activation of the diacylglycerol/Ras/extracellular signal-regulated kinase (ERK) cascade [38], which represents a pathway related to important vascular signaling effectors, such as angiotensin II or PDGF (platelet-derived growth factor). Although the physiological inhibitors of DGKs are not clear yet, recent studies show that the DGK isoforms possess two or three cysteine-rich domains essential for its full activity [38], which may render it susceptible to redox modifications of thiol groups. It is therefore possible that NO exposure promotes the release of exosomes from platelets by interfering in a similar pathway.

It must be pointed out that most of the studies concerning vascular signaling have been performed with a broader range of subcellular particles, known generically as microparticles. It is therefore difficult to perform comparisons and analysis of experimental results [39]. Different studies have shown that after interaction with target cells, platelet microparticles trigger some biological responses; for example, they activate endothelial cells [40], and induce [41] or inhibit the apoptosis of polymorphonuclear leukocytes [42]. In elegant studies, the group of M.Z. Ratajczak demonstrated that platelet microparticles could activate intracellular signaling pathways such as ERK and Akt, inducing angiogenesis and metastasis in lung cancer and promoting the survival and proliferation of normal human hematopoietic cells [32,43]. Nevertheless, the lipid, protein, or enzymatic species responsible for these effects could not be identified. Furthermore, studies by different groups have consistently demonstrated that circulating microparticles cause vascular dysfunction [44], impairing vasorelaxation and altering cardiac contractility in isolated vessel and heart models (L.C.P. Azevedo, unpublished data).

Although the mechanisms of vascular damage are not fully understood, they have been related to the generation of ROS [18]. In line with these results, in the present study we confirmed previous findings from our group demonstrating the presence of active NADPH oxidase and NO synthase in platelet-derived exosomes. Moreover, our data also suggest that a substantial portion of their redox-active properties could be attributed to the formation of the highly oxidative radical peroxynitrite.

To demonstrate that at least part of the proapoptotic activity of the exosomes could be related to the generation of ROS or RNS, we investigated the exosome-triggered SOD-mimetic, L-NAME, and urate inhibitable activation of caspase-3 in endothelial cells in culture. Caspase-3 activation and caspase-3-dependent apoptosis have been shown to be inhibited by S-nitrosation of a critical cysteine residue induced by exogenous NO donors [31]. Other studies, however, showed that caspase-3 (and caspase-2), as well as apoptosis, can be activated by exogenously added peroxynitrite [30]. In fact, NO has been implicated in regulating apoptosis in a variety of tissues [31]. In addition to the well established proapoptotic effects of NO [45], a growing body of evidence indicates that low levels of NO function as an important inhibitor of apoptosis by interference with signal transduction pathways that control apoptotic cell death [46]. In view of the ambivalent capacity of NO to act either as a proapoptotic or an antiapoptotic factor, closely related to the cell type and NO dosage, a complex spectrum of NO-mediated control of apoptosis is conceivable [47]. Thus, in accordance with the activation of NO synthases and with the cytosolic redox balance of the individual cell type in a given physiological scenario, NO may either function as an apoptotic inhibitor stabilizing tissue integrity or exert toxic effects.

## Conclusion

Taken together, our results confirm previous observations that exosome generation is a process subjected to specific regulatory pathways. In sepsis, both increased NO generation and the presence of LPS can trigger the release of platelet-derived exosomes, whereas thrombin or TNF-α induces the generation of phosphatidylserine-rich particles. Indicating an effective signaling role, septic-like platelet-derived exosomes induce caspase-3 activation and apoptosis of target endothelial cells through active ROS/RNS generation by NADPH oxidase and NO synthase type II. In addition, we propose that platelet exposure to LPS or NO *in vitro *may be a valuable model for the generation of exosomes involved in redox signaling.

Exosomes were first described in connection with the maturation of reticulocytes, and provide a method of sorting obsolete proteins, such as transferrin receptor, as the cells differentiate into erythrocytes. More recently, many other cell types have also been shown to secrete exosomes, such as antigen-presenting cells, which might use this mechanism to regulate the immune response. These findings prompted a reappraisal of the exosome's role from that of a 'garbage sack', releasing obsolete proteins, to a device involved in triggering intercellular communication. Here we propose that exosomes may have a major role in vascular redox signaling. In this context, exosomes could be a novel tool with which to further understand and possibly treat vascular dysfunction related to diabetes, hypertension, or sepsis.

## Key messages

• In sepsis, increased NO generation, as well as the presence of LPS, can trigger the release of platelet-derived exosomes.

• These exosomes induce caspase-3 activation and the apoptosis of endothelial cells through the generation of ROS and RNS.

• The existence of both specific releasing stimuli and targets characterizes exosomes as vascular signaling effectors.

• In sepsis, exosome generation may therefore contribute to endothelial dysfunction, leading to vascular failure.

## Abbreviations

DAF = 4,5-diaminofluorescein diacetate; DCHF = 2',7'-dihydrodichlorofluorescein diacetate; DGK = diacylglycerol kinase; D-NAME = *N*^ω^-nitro-D-arginine methyl ester; ERK = extracellular signal-regulated kinase; FITC = fluorescein 5(6)-isothiocyanate; L-NAME = *N*^ω^-nitro-L-arginine methyl ester; L-NMA = *N*^G^-methyl-L-arginine acetate; LPS = lipopolysaccharide; NO = nitric oxide; NOS = NO synthase (iNOS or NOS type II, inducible isoform; eNOS or NOS type III, constitutive isoform; nNOS or isoform type I, neuronal isoform); Nox1 and Nox2 = isoforms of membrane-bound subunits of NADPH oxidase; PBS = phosphate-buffered saline; PDI = protein disulfide isomerase; RNS = reactive nitrogen species; ROS = reactive oxygen species; SOD mimetic = membrane-permeable superoxide dismutase mimetic Mn(III) tetrakis (4-benzoic acid) porphyrin chloride; TNF = tumor necrosis factor.

## Competing interests

The authors declare that they have no competing interests.

## Authors' contributions

MHG performed Western blot and enzyme-linked immunosorbent assay studies and drafted the manuscript. AOC conducted the measurements of redox activity and apoptosis. LM participated in study design and performed all flow cytometry studies. SVF conducted Western blot studies as well as the electron microscopy. LRL participated in study design, coordination, and data analysis. MJ conceived of the study, participated in its design, coordination, and data analysis, and finished the manuscript. All authors read and approved the final manuscript.
